# Direct association with the vascular basement membrane is a frequent feature of myelinating oligodendrocytes in the neocortex

**DOI:** 10.1186/s12987-023-00425-4

**Published:** 2023-04-03

**Authors:** Justine S. C. Palhol, Maddalena Balia, Fernando Sánchez-Román Terán, Mélody Labarchède, Etienne Gontier, Arne Battefeld

**Affiliations:** 1 Univ. Bordeaux, CNRS, IMN, UMR 5293, Bordeaux, F-33000 France; 2 Univ. Bordeaux, CNRS, INSERM, Bordeaux Imaging Center, BIC, UAR 3420, US 4, Bordeaux, F-33000 France; 3 Present Address: Univ. Bordeaux, INSERM, Magendie, U1215, Bordeaux, F-33000 France

**Keywords:** Oligodendrocytes, Vasculature, Blood vessel-glia interaction, Gray matter, Mouse, SBF-SEM, Remyelination, Cuprizone, Whole-cell patch-clamp, Oligodendrocyte diversity

## Abstract

**Background:**

Oligodendrocyte lineage cells interact with the vasculature in the gray matter. Physical and functional interactions between blood vessels and oligodendrocyte precursor cells play an essential role in both the developing and adult brain. Oligodendrocyte precursor cells have been shown to migrate along the vasculature and subsequently detach from it during their differentiation to oligodendrocytes. However, the association of mature oligodendrocytes with blood vessels has been noted since the discovery of this glial cell type almost a century ago, but this interaction remains poorly explored.

**Results:**

Here, we systematically investigated the extent of mature oligodendrocyte interaction with the vasculature in mouse brain. We found that ~ 17% of oligodendrocytes were in contact with blood vessels in the neocortex, the hippocampal CA1 region and the cerebellar cortex. Contacts were made mainly with capillaries and sparsely with larger arterioles or venules. By combining light and serial electron microscopy, we demonstrated that oligodendrocytes are in direct contact with the vascular basement membrane, raising the possibility of direct signaling pathways and metabolite exchange with endothelial cells. During experimental remyelination in the adult, oligodendrocytes were regenerated and associated with blood vessels in the same proportion compared to control cortex, suggesting a homeostatic regulation of the vasculature-associated oligodendrocyte population.

**Conclusions:**

Based on their frequent and close association with blood vessels, we propose that vasculature-associated oligodendrocytes should be considered as an integral part of the brain vasculature microenvironment. This particular location could underlie specific functions of vasculature-associated oligodendrocytes, while contributing to the vulnerability of mature oligodendrocytes in neurological diseases.

**Supplementary Information:**

The online version contains supplementary material available at 10.1186/s12987-023-00425-4.

## Background

Interactions between oligodendrocyte lineage cells and the brain vasculature have been previously identified. During embryonic development, oligodendrocyte precursor cells (OPCs) migrate along the vasculature scaffold in the brain and spinal cord [1]. Similarly, attachment and migratory movements of OPCs are observed in adults in lysolecithin-induced lesions undergoing remyelination [2]. Endothelial cells secrete transforming growth factor beta 1 (TGF-β1), brain-derived neurotrophic factor (BDNF) and fibroblast growth factor (FGF) that all can influence differentiation and survival of OPCs [3, 4]. On the other hand, OPCs can stimulate angiogenesis during development and postnatal myelination through an HIF-activated secretion of Wnt and vascular endothelial growth factor (VEGF), presumably to adapt local blood supply to the metabolic needs during OPC differentiation [5, 6]. OPCs also participate in maintaining the blood-brain barrier integrity by secreting TGF-β1, which increases tight-junction protein expression in endothelial cells [7]. Moreover, in pathological tissue from multiple sclerosis patients, as well as in a mouse model, blood-brain barrier disruption occurs as consequence of aberrant OPC clustering around blood vessels [2], further suggesting that vasculature-OPC interaction is tightly regulated.

Similarly, the existence of functional interaction between mature oligodendrocytes and the vasculature has been established, but is not well explored. Endothelial cell released endothelin activates g-protein coupled endothelin receptor B on mature oligodendrocytes, subsequently stimulating myelin formation in the pre-frontal gray matter [8]. Conversely, in a model of white matter lesions, oligodendrocyte secreted metalloproteinase-9 can induce vascular remodeling, evidence of a direct influence of mature oligodendrocytes on the vasculature [9]. A possible functional connection between mature oligodendrocytes and the vasculature has been noted since the first description of oligodendroglia by Pio del Rio Hortega [10]. Vasculature-associated oligodendrocytes were found in white and gray matter of different species and a role in blood flow control by these oligodendrocytes was proposed [11] and recently associations of oligodendrocytes with blood vessels have been noted in the hippocampus [12]. More evidence that oligodendrocytes associate tightly with the vasculature emerged, after a cluster of presumably vessel-associated oligodendrocytes was identified in a single cell sequencing dataset analyzing cells of the brain vasculature [13]. Although these studies point to an interaction of mature oligodendrocytes with the vasculature, surprisingly little is known about occurrence and underlying structural properties of this cellular interaction in the adult neocortex.

In this study, we investigated the arrangement and frequency of oligodendrocyte-vasculature interaction in the neocortical gray matter. We found that vasculature-associated oligodendrocytes (vOLs) are common and are tightly associated with the endothelial basement membrane, suggesting that oligodendrocytes should be considered as an integral part of the vasculature microenvironment.

## Methods

### Animals

Mice were purchased from Charles-River or bred in-house. We used male and female C57Bl6/J or Cnp-mEGFP mice (Jackson Lab 026105, RRID:IMSR_JAX:026105 [14]) that were between 6 and 12 weeks old. Mice had unlimited access to food and water and were housed in individually ventilated cages on a standard light/dark cycle of 12/12 h. No statistical method was used to pre-determine sample size.

### Cuprizone administration

Male and female mice were fed for 5 weeks with powdered chow alone (control) or powdered chow supplemented with 0.2% cuprizone (bis-(cyclohexanone)-oxaldihydrazone, C9012, Sigma-Aldrich). Cuprizone-supplemented powder food was replenished every two to three days and supplied in powder food dispensers. After 5 weeks of cuprizone treatment, mice were either sacrificed (n = 6 mice, average age 12 weeks) or returned to a normal food pellet diet for 7 weeks (n = 4 mice, average age 22 weeks). For these two sets of experiments we used age matched control mice (demyelination: n = 3; remyelination: n = 4). Mice from these experiments were sacrificed as described in the following sub-section.

### Tissue preparation for immunohistochemistry

For all mice, anesthesia was induced with 3% isoflurane followed by intraperitoneal injection of a ketamine (100 mg/kg)/ xylazine (20 mg/kg) mix. Once mice were deeply anesthetized, we performed transcardial perfusion with PBS supplemented with heparin (12.5 U/ml), followed by freshly prepared 4% paraformaldehyde (Sigma-Aldrich). After fixation, brains were removed and post-fixed in 4% paraformaldehyde. In initial experiments post-fixation was for 24 h, but shortened subsequently to 2 h to improve antibody labeling as long post-fixation times masked some target antigens. All brains from cuprizone treated mice and respective controls were post-fixed for 2 h. Coronal slices of 50 μm nominal thickness were cut on a vibratome (VT1000S, Leica Microsystems, Germany) or on a freezing microtome (SM2010R, Leica). For cutting on a freezing microtome, all brains were cryoprotected by incubation in 15% sucrose solution in PBS at 4ºC followed by incubation in 30% sucrose solution in PBS at 4ºC. Thin sections were stored at 4 °C in PBS supplemented with 0.01% NaN_3_ for short-term storage or at − 20ºC in cryoprotective solution for long-term storage (30% glycerol and 30% ethylene glycol in PBS).

### Immunohistochemistry

For immunolabeling, we selected sections of the primary motor cortex and the primary somatosensory cortex (limb region) based on gross anatomical features and mouse brain atlases. All steps were conducted at room temperature (RT). Free-floating sections were incubated in blocking buffer consisting of 2.5% goat serum, 2.5% bovine serum albumin, and 0.3% triton X-100 in PBS for 1 h. Primary antibodies (see Table 1) were added to the blocking buffer and sections were incubated overnight. After washing with PBS, sections were subsequently incubated with secondary antibodies for 2 h in blocking buffer. Sections were washed with PBS and incubated for 5 min with 300 nm DAPI (Sigma-Aldrich) and washed afterwards. Tissue sections were mounted on glass slides with Fluoroshield mounting medium (F6182, Sigma-Aldrich) and cover-slipped.

**Table 1:** Antibodies used in this study

Antibody and dilutions	Manufacturer	Identifiers (Order number, RRID)
**Primary Antibodies**		
Rabbit polyclonal anti-CD31 (1:200)	Abcam	Cat #: ab28364, RRID:AB_726362
Rat monoclonal anti-CD13, cloneR3-63 (1:250)	Bio-Rad	Cat #: MCA2183T RRID:AB_1100679
Mouse monoclonal anti-CNPase, 11-5B, (1:500)	Sigma-Aldrich	Cat #: C5922, RRID:AB_476854
Mouse monoclonal anti-myelin basic protein (MBP), clone SMI 99, (1:500)	Biolegend	Cat #: 808,402, RRID:AB_2564742
Rabbit polyclonal anti-Collagen-IV biotinylated (1:500)	Abcam	Cat #: ab6581, RRID:AB_305579
Rat monoclonal anti-Transferrin Receptor (1:500)	Novus Biologicals	Cat #: NB100-64979, RRID:AB_962622
Rabbit polyclonal anti-AQP4 (1:5000)	Sigma-Aldrich	Cat #: HPA014784, RRID:AB_1844967
Mouse recombinant anti-GFP [N86/38.1R] (1:5)	cell-culture supernatant, laboratory	gift from James Trimmer Addgene plasmid # 114,492, RRID:Addgene_114492
**Secondary Antibodies**		
Goat polyclonal anti-rabbit Alexa 488 (1:500	Invitrogen	Cat#: A11034, RRID:AB_2576217
Goat polyclonal anti-mouse IgG2A Alexa 488 (1:500)	Invitrogen	Cat#: A21131, RRID:AB_2535771
Goat polyclonal anti-mouse IgG1 Alexa 488 (1:500)	Invitrogen	Cat#: A21121, RRID:AB_2535764
Goat polyclonal anti-rat Alexa 555 (1:500)	Invitrogen	Cat#: A21434, RRID:AB_2535855
Goat polyclonal anti-rabbit Alexa 568 (1:500)	Invitrogen	Cat#: A11036, RRID:AB_10563566
Goat polyclonal anti-mouse IgG1 568 (1:500)	Invitrogen	Cat#: A21124, RRID:AB_2535766
Goat polyclonal anti-rabbit Alexa 647 (1:500)	Invitrogen	Cat#: A21245, RRID:AB_2535813
Goat polyclonal anti-rabbit IgG Atto-647 N (1:500)	Sigma-Aldrich	Cat#: 40,839 RRID:AB_1137669
Goat polyclonal anti-mouse IgG1 Alexa 647 (1:500)	Invitrogen	Cat#: A21240, RRID:AB_2535809
**Streptavidins and Lectin**		
Streptavidin Alexa Fluor 555 (1:500)	Invitrogen	Cat#: S21381, RRID:AB_2307336
Streptavidin Alexa Fluor 594 (1:500)	Jackson Immuno Research	Cat#: 016-580-084 RRID:AB_2337250
Lycopersicon Esculentum (Tomato) Lectin DyLight-649 (1:1000)	Vector Laboratories	Cat #: DL-1178-1

### Confocal microscopy

Microscopy images were acquired with Leica TCS SP5 confocal microscopes (Leica Microsystems, Germany) controlled by Leica Application Suite software. Tiled z-stack scans covering all neocortical layers were acquired either with a 40 × 1.30NA objective (HC PL APO CS2 OIL UV, Leica) or a 40 × 1.25NA objective (HCX PL APO lambda blue OIL UV, Leica). Higher magnification z-stacks of individual oligodendrocytes located in apposition to blood vessels were taken with a 63 × 1.40NA objective (HCX PL APO CS OIL UV, Leica).

### Deconvolution of high-resolution images

High-resolution image stacks were deconvolved using the AutoQuant 3D deconvolution module (version X3.1.2, RRID:SCR_002465, Media Cybernetics, Rockville, MD, USA). We used the adaptive point-spread function method with a theoretical point-spread function and performed 4 iterations with the noise level set to medium.

### Tissue fixation for electron microscopy

For electron microscopy (EM), nine mice were used to optimize the parameters for serial block-face imaging, including perfusion, tissue fixation, tissue preparation, fluorescence conservation, blood-vessel labeling and laser marking. For our final experiments, we performed transcardial perfusion of two mice with PBS (10–15 ml) supplemented with heparin (12.5 U/ml) followed by 25 ml RT fixative solution consisting of 4% PFA and 2.5% glutaraldehyde (both Electron Microscopy Science, Hatfield, PA, USA) in PBS at a flow rate of 2 ml/min. After perfusion the brains were carefully removed from the skull and post-fixed for 24 h in the same fixative and then washed several times in PBS before sectioning. Coronal sections of the brains were cut on a VT1000S vibratome (Leica) to a nominal thickness of 100 μm and stored in 0.1 M phosphate buffer (PB). Slices were washed with PB and stored in PB supplemented with 0.02% NaN_3_ until further processing steps.

### 2-photon near-infrared branding for serial block-face imaging

As conventional immunohistochemistry methods do not preserve the ultrastructure, we took advantage of the oligodendrocyte reporter mouse that expresses membrane- tethered EGFP in all oligodendrocyte processes and cell bodies under the Cnp promoter. Preliminary experiments confirmed that EGFP fluorescence was preserved after fixation of tissue for EM. Labeling of the vasculature was achieved by incubating 100 μm slices overnight with tomato lectin conjugated with DyLight-649 (Table 1) in PB, which did not require permeabilization. Sections were washed multiple times with PB and transferred to a glass petri dish filled with PB and held with a slice anchor (SHD-27 H/15, Warner Instruments, Holliston, MA, USA) for easy recovery of the slices after near-infrared branding. All procedures were performed on a SP5 confocal microscope (Leica) controlled by LAS software (Leica) and equipped with a 25 × 0.95NA water-dipping objective (HCX IRAPO, Leica). We performed overview scans at 2048 × 2048 pixels (zoom 1 to 1.5) to identify vOLs and subsequently marked several vOL positions in each slice with laser burns.

For burning laser marks, we used a tunable pulsed 2-photon laser (Mai Tai HP, Spectra Physics/Newport, Irvine, CA, USA) with an average output of ≈ 1.8 W and wavelength set to 910 nm. 2-photon laser output power (transmission) was not attenuated and kept at 100%, gain was set to 100% and the offset to 73%.

For each identified vOL, the oligodendrocyte cell body was centered under a small horizontal burn mark of ≈ 20 μm length (XZT mode; digital zoom 30; 512 × 32 pixels/image; 200 Hz scan speed; 50 lines; average pixel dwell time 10 µs). To identify the position of the laser marks during EM sample preparation, we burned a 300 μm long horizontal line (XZT mode; digital zoom 2; 512 × 32 pixels/image; 200 Hz; 1000 lines) and a large square (XYT mode; digital zoom 15; 64 × 64 pixels/image; 50 Hz; 300 frames) above the regions of interest (ROIs). Repetitions were set to match required thickness and line depth (Supplementary Fig. 3B,C). Location, width and depth of branding marks were subsequently imaged using a 561 nm laser in normal confocal scan mode, taking advantage of tissue auto-fluorescence on the borders of laser marks.

### Sample preparation for block-face electron microscopy

Tissue for serial block-face scanning electron microscopy (SBF-SEM) was prepared similar as previously described [15]. Samples were post-fixed with a 2% paraformaldehyde and 2.5% glutaraldehyde solution (Electron Microscopy Science) in 0.15 M cacodylate buffer (pH 7.4) for 2 h at RT and subsequently washed five times for 3 min in cold 0.15 M cacodylate buffer. To enhance contrast of the tissue, we performed consecutive incubations in heavy metal solutions. Samples were incubated for 1 h in 2% osmium tetroxide containing 1.5% potassium ferrocyanide in 0.15 M cacodylate buffer on ice. After washing five times for 3 min in ultrapure water, the samples were incubated for 20 min in a freshly prepared thiocarbohydrazide solution (1% w/v in water) at RT. Samples were washed five times for 3 min in ultrapure water and then incubated in 2% osmium tetroxide in water at RT for 30 min. After washing 5 times for 3 min in ultrapure water the tissue was incubated in 2% uranyl acetate at 4 °C overnight. Finally, Walton’s lead aspartate staining was performed for 30 min at 60 °C. We prepared a fresh 30 mM L-aspartic acid solution to dissolve lead nitrate 20 mM, pH 5.5), which was subsequently filtered to remove undissolved particles. After final washing steps in ultrapure water, the samples were dehydrated in ice-cold solutions of 30%, 50%, 70%, 90%, and twice in 100% ethanol (anhydrous), and twice in 100% acetone for 10 min each. Tissue was embedded in epon by placing the samples in 25% acetone/epon for 2 h, 50% acetone/epon for 2 h, 75% acetone/epon for 2 h and followed by two incubations in 100% epon (overnight, 8 h). The samples were then transferred to fresh epon resin fand incubated at 60 °C for 48 h. Once the resin blocks were hardened, blocks were coarsely cut with a razor blade to generate a pyramidal shaped sample. Embedded samples were mounted on aluminum specimen pins using a silver filled conductive resin (Epotek-Delta microscopies, Mauressac, France). After 24 h of polymerization at 60 °C, the samples were further trimmed with a diamond knife (Diatome, Nidau, Switzerland) on an ultramicrotome (Leica EM UC7). Silver filled conductive resin was used to electrically connect the edges of the tissue to the aluminum pin. The entire sample was sputter-coated with a 5–10 nm layer of gold to enhance conductivity.

### Serial block-face scanning electron microscopy (SBF-SEM) for 3D EM

SBF-SEM was performed with a ZEISS Gemini field emission gun SEM300 (Zeiss, Marly-le-Roi, France), equipped with a 3View2XP in situ ultramicrotome (Gatan Inc., Pleasanton, CA, USA). Serial section thickness was set to 30 nm. The block-face was imaged with the accelerating voltage set to 1.2 kV and backscattered electrons were detected with an OnPoint Detector (Gatan Inc.) and a pixel dwell time of 6 µs. Images were acquired in high vacuum mode with magnifications and image sizes adjusted to a final pixel size of 10 nm at specimen level.

### Microns Explorer dataset

We used the large scale publicly available electron microscopy IARPA MICrONS dataset of a 12 week old mouse visual cortex [16] to further analyze the ultrastructure of oligodendrocyte vasculature arrangements. All elements in this dataset were segmented by a machine learning approach from the consortium. We identified oligodendrocytes by their morphology with several short processes that we then traced to myelin segments as myelin was not segmented. We identified 5 vOLs within the volume and subsequently scanned the z-section for contact sites of oligodendrocytes with the vasculature. The automatic segmentation was not always precise overestimating the contact sites or providing wrong annotations. Analyzed oligodendrocytes and blood vessels and their corresponding IDs are summarized in Supplementary Table 1.

### Electrophysiology

For electrophysiological investigations of oligodendrocytes we used Cnp-mEGFP reporter mice aged 43 ± 7 days. Mice were deeply anesthetized with a mixture of ketamine (100 mg/kg) and xylazine (20 mg/kg). For some animals, we performed transcardial perfusion with ice-cold preparation solution before decapitation, however, blood vessel visibility was reduced in acute slices. In a subset of experiments, we therefore omitted transcardial perfusion and decapitated deeply anesthetized mice followed by quick removal of the brain. The brain was then submerged in ice-cold preparation solution saturated with 95% O^2^, 5% CO^2^ carbogen composed of (in mM) 60 NaCl, 25 NaHCO_3_, 1.25 NaH_2_PO_4_, 2.5 KCl, 100 sucrose, 1 CaCl_2_, 5 MgCl_2_ and 20 glucose. Subsequently, we cut 300 μm para-sagittal slices of the neocortex on a vibratome (VT1200S, Leica, Germany). Slices were collected in carbogen-saturated storage solution composed of (in mM) 125 NaCl, 25 NaHCO_3_, 1.25 NaH_2_PO_4_, 3 KCl, 1 CaCl_2_, 6 MgCl_2_ and 20 glucose. Slices were incubated for 35 min at 35ºC before being kept in the same solution at RT for the experimental day.

For recordings, slices were transferred to a heated (32 ± 1ºC) submerged recording chamber on an upright microscope (Olympus or LN-scope) equipped with infra-red illumination and oblique contrast optics for visualization of cells. The recording solution was composed of (in mM) 125 NaCl, 25 NaHCO_3_, 1.25 NaH_2_PO_4_, 3 KCl, 2 CaCl_2_, 1 MgCl_2_ and 25 glucose. Oligodendrocytes were identified in the somatosensory cortex of CNP-mEGFP mice by epifluorescence illumination with a 470 nm LED and a GFP filter set (GFP-30LP-B-000, Semrock, Rochester, NY, USA). Vasculature-associated oligodendrocyte cell bodies were identified by switching between epifluorescence and infra-red illumination.

For whole-cell recordings, we used borosilicate glass pipettes (BF150-86-10, Sutter Instrument, Novato, CA, USA) pulled on a vertical puller (PC100, Narishige International Limited, London, UK) to a size of ≈ 5–7 MΩ. Pipettes were filled with intracellular solution consisting of (in mM) 130 K-Gluconate, 10 KCl, 10 HEPES, 4 Mg-ATP, 0.3 Na2-GTP, 10 Na_2_-phosphocreatine with a pH set to 7.25 with KOH and an osmolarity adjusted to 280 mOsm. All satellite oligodendrocytes and 5 vOLs were recorded with a modified intracellular solution containing 125 mM K-Gluconate and additionally 20 µg/ml glycogen and 0.32 U/µl Ribolock (Thermo-Scientific). The lowered potassium concentration in the latter solution accounted for changes in osmolarity introduced by Ribolock. Using the Nernst equation we estimated the potassium equilibrium potential to be -101.39 mV in the standard solution and − 100.42 mV in the modified solution resulting in a 0.96 mV calculated difference. All membrane parameters for vOLs that were recorded with either solution were the same (Mann Whitney tests) thus we subsequently pooled all vOL data. Experiments were controlled, and acquired by an integrated patch-clamp amplifier system with SutterPatch software (IPA2, Sutter Instruments, Novato, CA, USA). Recordings were sampled with a minimum frequency of 10 kHz and filtered with a 4-pole Bessel filter set to 5 kHz. After establishing whole-cell configuration in voltage-clamp, we performed recording of membrane potential and membrane characteristics in current-clamp.

## Quantifications and analysis

### Image analysis software

All image analysis was carried out with FIJI [17], Digital Micrograph software (Gatan Inc., Pleasanton, CA, USA), Microscopy Image Browser – MIB v.2.702 (RRID:SCR_016560 [18]), or Imaris version 9.8.0 (Oxford Instruments, Belfast, UK). Imaris was also used to generate 3D reconstruction of images.

### Oligodendrocyte-vasculature distance measurement

**Fig. 1:**
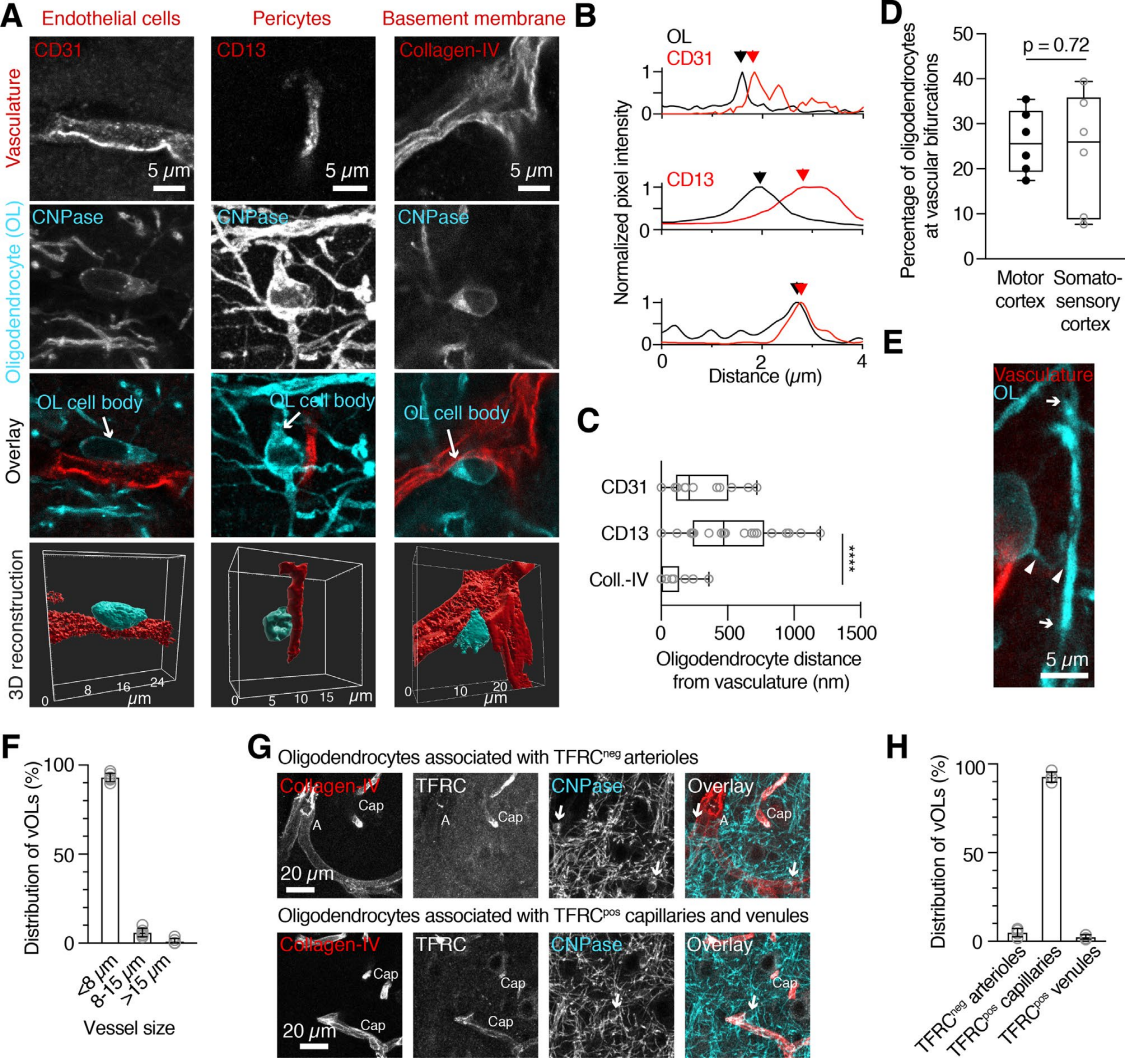
**Oligodendrocytes are closely and predominantly associated with capillaries in the neocortical gray matter** (A) Representative images of vOLs and the major vascular components: endothelial cells (CD31), pericytes (CD13) and basement membrane (collagen-IV), and corresponding 3D reconstructions. Oligodendrocyte cell bodies (cyan) appear directly associated with the vasculature (red). (B) Normalized pixel intensity plots used for quantifying the distance between vasculature and oligodendrocytes. Labeling of either CD31, CD13 or collagen-IV shows sub-micrometer distances between blood vessel components and oligodendrocyte cell body. Maximum peaks of the immunosignal (red and black arrowheads) were used to quantify spacing between immunosignals. (C) Quantification based on (B) reveals the shortest distance for collagen-IV compared to CD31 and CD13, consistent with blood vessel anatomy (Kruskal-Wallis test, p = 0.0001, n = 12 cells for CD31, n = 17 cells for collagen-IV, n = 29 cells for CD13, each from n = 4 mice). (D) Quantification of oligodendrocytes on vascular bifurcations from the total population of vOLs (Unpaired t-test, p = 0.72, n = 6 mice/area). (E) High magnification image of a myelin sheath connected to a vOL. Arrowheads indicate the cell process linking the myelin sheath to the cell body, arrows mark the extension of the internode. The majority of the blood vessel was cut for display. (F) Distribution of vOLs on different sized blood vessels shows that the majority is associated with vessels < 8 μm, presumably capillaries (n = 6 mice). (G) Representative images of oligodendrocytes on transferrin receptor negative (arterioles) and positive (capillaries and venules) blood vessels. Arrows point to oligodendrocyte cell bodies. A: arteriole, Cap: capillary. (H) Quantification of blood vessel types reveals that the majority of vOLs are located on transferrin-positive capillaries (n = 4 mice).

The distance separating the oligodendrocyte cell body and blood vessel wall (Fig. 1D) was estimated from deconvolved high-resolution images using FIJI. The fluorescence intensity of each channel (CNPase, CD13 and CD31 or Collagen-IV) was measured along a line crossing the contact site (line tool) and the distance between the fluorescence peaks was calculated.

### Oligodendrocyte-blood vessel contact area analysis

For accurate reconstructions of oligodendrocyte cell bodies, we segmented the soma outline from deconvolved high-resolution images using MIB software. We exported and rendered the created model as an Imaris surface element and combined the model with the source image in Imaris. The blood vessel surface was reconstructed using the automatic surface creation module of Imaris (settings: smooth; surface detail: 0.120 μm; thresholding: absolute intensity). The surface of contact between the oligodendrocyte soma and blood vessel was created and measured with the surface-surface contact area Imaris XTension (https://imaris.oxinst.com/open/view/surface-surface-contact-area). We set the oligodendrocyte surface as primary surface to avoid an overestimation of the contact area.

### Aquaporin-4 intensity measurements

AQP4 fluorescence was measured from confocal images with line or area ROIs (FIJI) that were placed on AQP4 positive blood vessels, either at the oligodendrocyte-vasculature contact site or outside the contact site.

### Oligodendrocyte number, distribution and density

Neocortical organization of oligodendrocytes (Figs. 1, 3 and 5) was analyzed on image z-stacks in FIJI. For each control mouse (n = 6) images were taken from 4 or more independent sections. We quantified on average 0.052 ± 0.01 mm^3^ from 3 or more fields of view (on average 5 ± 0.25 fields per mouse). For demyelination (n = 6 mice) and remyelination (n = 4 mice) as well as age matched controls (n = 3 and n = 4, respectively), we analyzed between 4 and 6 different fields of view of at least 2 sections with an average volume of 0.9*10^6^ µm^3^. Oligodendrocyte locations in the cerebellum (n = 3 mice) and hippocampus (n = 4 mice) were determined from 3 images per mouse (average volume of 3*10^6^ µm^3^)  located in 3 independent slices. When applicable, we averaged the results from all images for each animal, to have one data point for statistical analysis.

For layer distribution analysis, a grid with each individual square being 25 µm by 25 µm was overlayed and aligned to the surface of the cortex. Oligodendrocytes were manually counted and their coordinates were registered using the point tool and ROI Manager. The layers of the neocortex were determined based on distance from the pia and labeling of DAPI and CNPase, reflecting cell and myelin densities respectively. An oligodendrocyte was considered as vasculature-associated when the distance separating its cell body from blood vessel wall (measured as described above) was ≤ 1 μm. The same analysis workflow was applied for data presented in Figs. 1, 3 and 5.

To assess the randomness of oligodendrocyte distribution we processed the images as follows. The single channels of the z-stacks were separated in FIJI using the “Split Channels” command. Next, the channel containing the vasculature labeling was transformed with the “flip horizontally” command. Finally, the separated channels of the vasculature and oligodendrocyte labeling were re-combined into a multi-channel tif image and subsequently analyzed as described above.

### Blood vessel diameter

For each vOL identified in image z-stacks, the diameter of the associated blood vessel was measured with the line tool (1 pixel width, FIJI) within a maximum 20 μm radius around the vOL cell body. Three categories of vessels were defined based on their diameter: <8 μm (cerebral capillaries), 8 to 15 μm (precapillary arterioles or postcapillary venules) and > 15 μm (arterioles orvenules and arteries or veins) [19, 20].

### Analysis of myelin and vasculature density

ROIs of 10^4^ µm^2^ were delimited in each neocortical layer from the same images that were used for oligodendrocyte quantification. All analysis was performed with FIJI.

Myelin density: A threshold was set for single z-plane images containing myelin labeling (CNPase) and the image was subsequently de-speckled to remove 1 × 1 positive pixels. The total myelin occupying pixels were measured and myelin density was calculated by dividing the myelin positive area by the total area of the ROI.

Vasculature density: We performed maximum intensity z-projections of images with collagen-IV labeling. An image threshold was set and positive outliers (< 3 pixels) resulting from image acquisition noise were removed. Vasculature density was calculated by dividing the total blood vessel area by the volume of the ROI.

### Image processing of electron microscopy images

Automatic image registration and alignment of the raw image stack was performed with Digital Micrograph using the forward running image as reference and a subpixel alignment accuracy to remove drift from image acquisition. The resulting stack was cropped to an area common for all images. All further image processing was performed on tif images. We used MIB for manual segmentation of the blood vessel lumen, basement membrane, oligodendrocyte cell body and main protruding processes using a drawing tablet and pen (Cintiq pro 5, Wacom, Saitama, Japan) as input device. We created independent models of each element from which we generated 3D models in Imaris (9.8.0).

### Electrophysiological analysis

Data were exported from Sutter Patch and analyzed offline using pClamp 10.1 (Molecular Devices LLC, San Jose, CA, USA). The resting membrane potential (*V*_m_) was determined from baseline segments of current-clamp recording traces with zero-current injection. Input resistance (*R*_in_) was estimated by fitting the linear range of voltage responses to 400 pA step current injections ranging from − 800 to 400 pA. Resting conductance (*G*_m_) was calculated as *G*_m_ = ΔI/ΔV where ∆V was based on a 10 mV hyperpolarizing voltage step from holding potential. For current-voltage (I-V) relationship analysis, an evaluation of voltage dependence of underlying currents, we measured the steady-state current (indicated in Fig. 4D) as a response to voltage steps between − 10 mV and − 130 mV. Principal component analysis was performed by using the R [21] *prcomp* function with standard parameters and inclusion of four electrophysiological variables: resting membrane potential, conductance, input resistance and capacitance. All reported voltage values were corrected for a liquid junction potential of − 15 mV as previously determined [22].

### Statistical analysis

The details about experimental size and statistical tests that we performed are provided in the figure legends or in the main text. Data were statistically analyzed in Prism 9 (Version 9.3, GraphPad Software, San Diego, CA, USA). To determine the right statistical test, data were first tested for normal distribution with a Shapiro-Wilk test. For comparison between two groups, non-normally distributed data were analyzed by a Mann-Whitney test and normally distributed data were tested with either a paired or unpaired t-test. Comparisons of more than two experimental groups were performed using a non-parametric Kruskal-Wallis test as data were not normally distributed. A probability of p < 0.05 was considered statistically significant, exact p-values are provided in the figures, figure legends or in the text. Data are given as mean ± standard error of the mean.

## Results

### Oligodendrocytes associate with the vasculature in the neocortex

Early work noted the presence of oligodendrocytes close to blood vessels in the cortex [10, 11], but occurrence and precise location on the vascular network remained largely unexplored. Using immunohistochemistry, we labeled mature oligodendrocytes with an antibody against 2′,3′-cyclic nucleotide-3′-phosphodiesterase (CNPase) as this antibody permitted the detection of oligodendrocyte cell bodies in contrast to the myelin marker myelin basic protein (MBP, Supplementary Fig. 1). From confocal images we then determined oligodendrocyte location in relation to major components of the brain vasculature in 8-week-old mice. We labeled endothelial cells with the marker CD31, pericytes with the marker CD13 and the basement membrane with collagen-IV (Fig. 1A). Since vOLs were in close contact with the vasculature, we measured the distance between randomly selected vOL cell bodies and the different blood vessel components based on fluorescence intensity peaks for the respective markers (Fig. 1B). vOL cell bodies were furthest away from the pericyte marker CD13 (507 ± 340 nm, n = 29 vOLs, n = 4 mice) reflecting the non-homogeneous coverage of blood vessels by pericytes (Supplementary Fig. 2). The endothelial cell marker CD31 was in intermediate distance (300 ± 247 nm, n = 12 vOLs, n = 4 mice) and the basement membrane marker collagen-IV was closest (80 ± 127 nm, n = 17 vOLs, n = 4 mice, Fig. 1E). This short distance from the basement membrane indicated a possible direct contact with the vasculature. Additionally, we noted that the majority of oligodendrocyte cell bodies contacted blood vessels along unbranched sections (78%). From all quantified vOLs, 22% were located at a vascular branching site in both motor- and somatosensory cortex (309 out of a total of 1392 vOLs from 6 mice, Fig. 1A and D), which was independent of differences in vascular branching density [23]. Next, we traced processes from oligodendrocyte cell bodies labeled with CNP to MBP positive myelin sheaths, suggesting that vOLs are myelinating (n = 12 oligodendrocytes, n = 4 mice, Fig. 1E).

The vasculature is divided into arterial, capillary and venous networks, which differ in terms of size, structure and function. We asked whether vOLs preferentially locate to specific parts of the vascular network. Based on the blood vessel diameter at the contact site, the majority (93% ± 2.4%) of oligodendrocytes were located on vessels with a diameter < 8 μm (969 of 1049 cells, n = 6 mice, Fig. 1F) corresponding to capillaries (see methods for definition). Only a few oligodendrocytes were located on larger diameter vessels (> 8 μm 66 of 1049 cells; >15 μm 14 of 1049 cells). The relatively lower occurrence of vOLs on larger diameter vessels can be explained by the low density of larger penetrating arterioles and is in line with volumetric data from mouse cortex, showing that the majority of vessels are micro-vessels [20]. To determine if vOLs on larger vessels are found on the arterial or venous part of the vascular network, we next labeled blood vessels with transferrin receptor (Tfrc), which is expressed in capillaries and veins, but not in arteries [13]. Similar to the size distribution analysis, the majority of oligodendrocytes (254 of 284 vOLs, n = 4 mice) were located on Tfrc-positive capillaries. In addition, few oligodendrocytes were found on either Tfrc-positive venules or Tfrc-negative arterioles (30 out of 284 vOLs, n = 4 mice, Fig. 1G, H). Additionally, Tfrc labeling was absent from vOLs (n = 8 vOLs, n = 3 mice). In summary, our data establish that in the neocortex, vOL cell bodies are in close contact with different blood vessel types and mostly associate with capillaries.

**Fig. 2:**
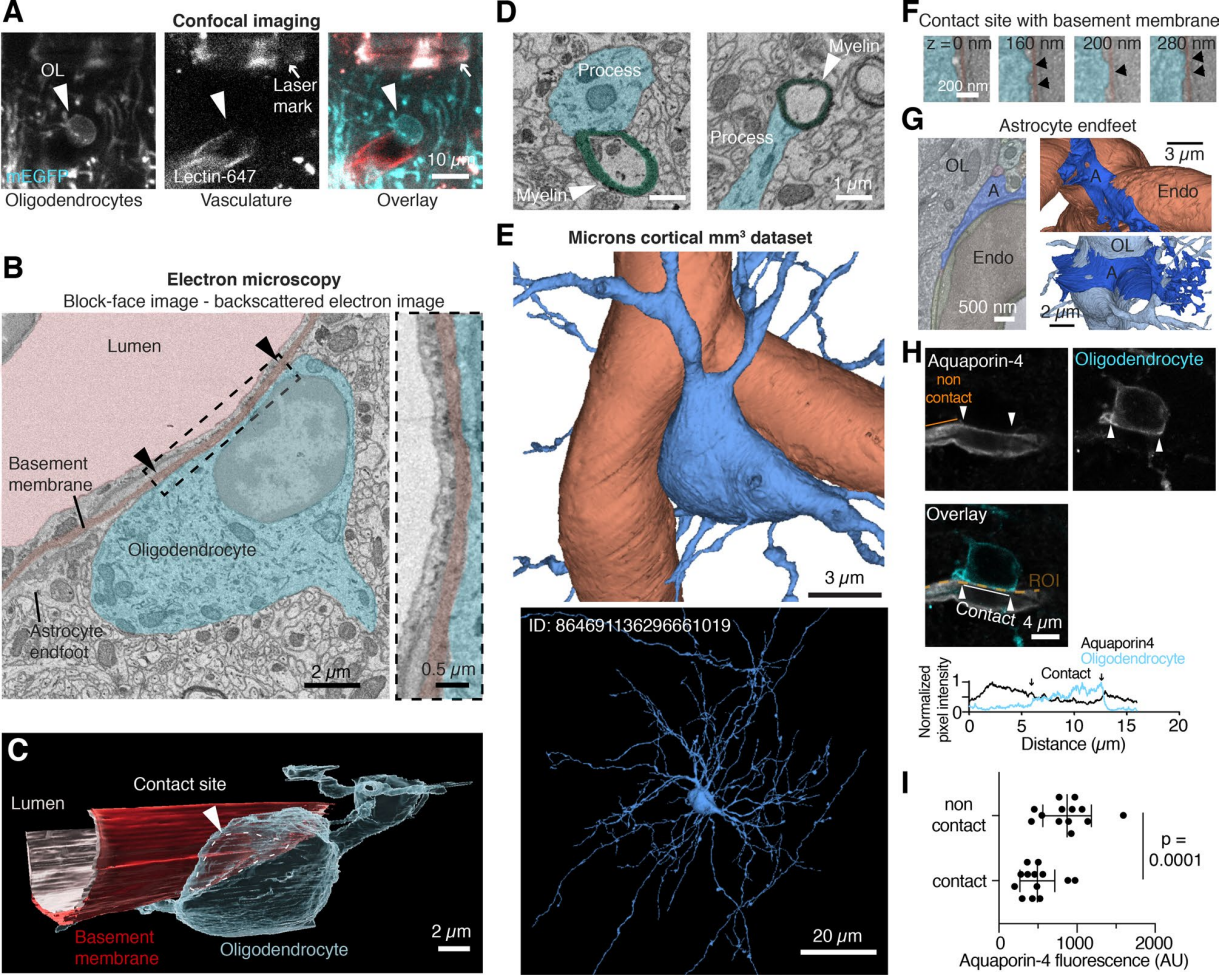
**Oligodendrocytes are in direct contact with the vascular basement membrane** (A) Confocal images of a vOL (cyan, arrowhead) on a blood vessel (red). The location was marked with 2-photon branding and the created laser mark is well visible using green excitation light (see arrow). (B) Single-plane block-face image of the same oligodendrocyte as in (A) showing the direct contact between the cell body (light cyan,) and the basement membrane (dark red). Arrowheads denote the length of the contact and the boxed area is displayed at higher magnification. (C) 3D rendering of the reconstructed vOL in A and B, the basement membrane and the blood vessel lumen. The oligodendrocyte cell body closely follows the blood vessel shape. (D) Two example EM images showing traced processes from the cell body (light cyan) ending in compact myelin (green). (E) Example 3D volume image of a vOL (blue) and vasculature (red) obtained from the Microns dataset. (F) Example EM false colored images of contact sites between the vasculature and vOLs (cyan) from the MICrONS dataset. Arrows point to the contact site. (G) Left: annotated example EM image of astrocyte endfeet (A), oligodendrocyte (OL) and a presumed endothelial cell of the vasculature (Endo). Right: 3D reconstructions of the microns dataset show the arrangement of the astrocyte endfeet on the vasculature (top) and oligodendrocyte (bottom). (H) Single plane confocal images showing aquaporin-4 labeling of the vasculature (gray) and oligodendrocyte cell body (cyan) labeling in a Cnp-mGFP mouse. Bottom: Fluorescence intensity plots for both channels, showing a reduction of Aquaporin-4 labeling intensity at the contact site. (I) Quantification of the aquaporin-4 fluorescence and comparison between contact and non-contact sites show a reduced intensity of aquaporin-4 signal at the contact sites (Paired t-test, p = 0.0001, n = 13 cells from 4 animals).

### Oligodendrocytes directly contact the vascular basement membrane

Although our immunohistochemistry experiments revealed that oligodendrocytes are in close contact with the vasculature, the measured distance was at or below the resolution limit of standard optical microscopy. We therefore investigated this contact further with electron microscopy (EM) to visualize the ultrastructure at nanometer resolution. To specifically target vOLs, we used a correlative light and EM approach, in which we marked the tissue location of vOLs with 2-photon near-infrared branding (Fig. 2A and Supplementary Fig. 3A-G) ahead of sample preparation for EM [24]. From confocal images we determined that the targeted oligodendrocytes were located at small diameter blood vessels (6.9 ± 0.8 μm, n = 4 vessels, 1 vOL was at a bifurcation and therefore close to 2 vessels, n = 2 mice), presumably capillaries, which were found at an average depth of 680 ± 124 μm (n = 3 vOLs) from the pia, corresponding to the border of neocortical layers 4 to 5. We then re-identified the laser-tagged regions in EM and subsequently imaged volumes of the tissue containing the vOLs with serial block-face scanning electron microscopy (SBF-SEM). Analysis of these images established that the vOL cell body is in direct contact with the basement membrane throughout several z-sections (n = 3 vOLs from 2 mice, Fig. 2B). A 3-dimensional reconstruction (Fig. 2C) showed that the oligodendrocyte soma and the vascular basement membrane are in direct contact over a large area, surrounded by probably astrocytic endfeet. As we targeted small diameter blood vessels, representing capillaries, our results may not apply to vOLs associated with other blood vessel types. Processes protruding from the cell body of the imaged vOLs were contacting compact myelin in adjacent serial sections (Fig. 2D), confirming that vOLs myelinate axons. The cytoplasm of the oligodendrocyte cell body was darker than surrounding tissue elements (Supplementary Fig. 3H), in line with previous observation [25, 26] and only a thin layer of cytoplasm surrounded the nucleus that was located to one side of the cell. We could further identify Golgi apparatus, microtubules, endoplasmic reticulum, mitochondria and lipid inclusions in the vOLs (Supplementary Fig. 3I-M). To estimate the contact area, we used confocal images of vOLs and blood vessels. The average contact area was 36.5 ± 23.1 µm^2^, representing about 13.6 ± 6.7% (n = 13 vOLs, n = 3 mice) of the total oligodendrocyte soma surface area.

Moreover, we identified and analyzed 5 additional vOLs in a large-scale segmented 3D electron microscopy dataset [16] of the visual cortex (Fig. 2E). This analysis confirmed at least 2 direct contact sites with the vascular basement membrane (Fig. 2F). Contact sites of all ensembles were small and short and stretched over several z sections. As all elements in the dataset were segmented, we could also identify that at non-contact sites a thin sheet of astrocytic endfeet measuring from 10 to 50 nm were between the oligodendrocyte and the vasculature (Fig. 2G).

We additionally labeled endfeet with aquaporin-4 (AQP4, Fig. 2H) and assessed the presence of AQP4 labeling at or around the contact site with confocal microscopy. We observed a lower fluorescence intensity of AQP4 at the contact site, compared to the blood vessel segments that were not in contact with the oligodendrocyte (paired t-test, p = 0.0001, n = 13 vOLs, n = 4 mice, Fig. 2G,H). This result suggests that astrocytic endfeet are contacting vOL cell bodies, but are strongly reduced in size or absent around the contact site as demonstrated by the two electron microscopy datasets. We conclude that vOLs are in immediate proximity to astrocytic endfeet that surround the vasculature and that vOLs establish a direct contact with the vascular basement membrane.

### Distribution and physiological properties of vasculature-associated oligodendrocytes in the neocortex

**Fig. 3:**
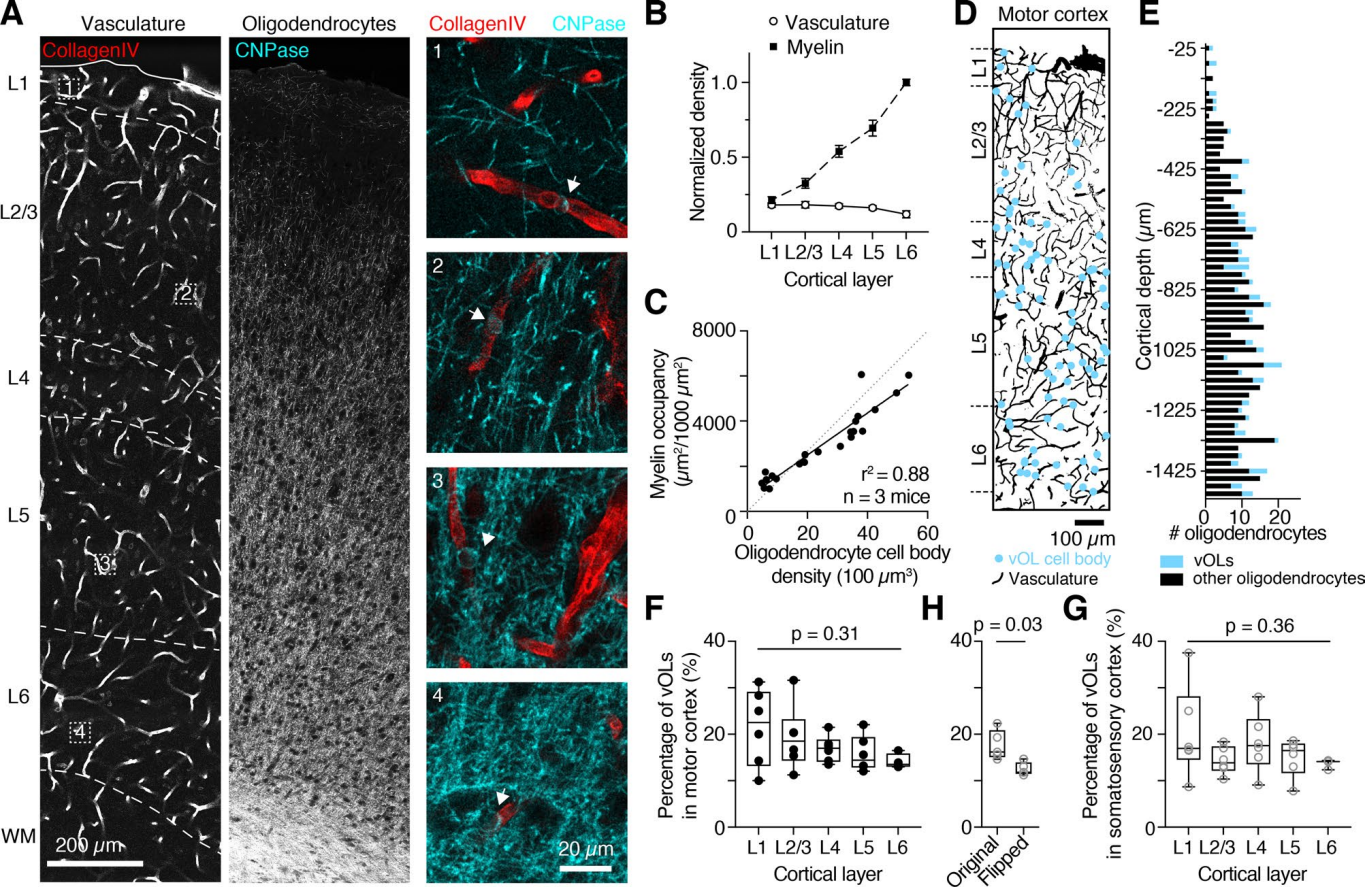
**Oligodendrocytes associate with the vasculature in all neocortical layers** (A) Representative single plane confocal images of motor cortex showing collagen-IV labeled vasculature (left, red) and CNPase labeled oligodendrocytes (center, cyan). On the right, four example vOLs from Layer 1, 2, 4 and 6 are displayed at higher magnification and their locations are indicated with arrows and corresponding numbers on the left image. (B) Quantifications reveal that vascular density remains constant throughout the cortex, whereas myelin density increases towards deeper layers. Data were normalized to myelin density in layer 6 (n = 3 mice). (C) Oligodendrocyte cell body density is highly correlated with myelin immunosignal (myelin occupancy), confirming that oligodendrocyte cell body location and density can be used to infer myelination density. Data were fit with a linear function (n = 3 mice). (D) Vasculature (black) from a z-projected cortex volume (same as in A) overlayed with vOL locations (cyan). (E) Plot showing oligodendrocytes occurrence within the volume in D (25 μm bins). Vasculature-associated oligodendrocytes are highlighted in cyan. (F, G) Throughout all neocortical layers, the proportion of vOLs remains constant in the motor cortex (F) and somatosensory cortex (G). Data were tested with a Kruskal-Wallis test and p value is given in the figure (n = 6 mice for layers 1 to 5, and n = 3 mice for layer 6). (H) Random distribution of oligodendrocytes resulted in a lower percentage of vOLs in the same area.

Since oligodendrocytes are non-uniformly distributed across the cortex [27], we asked whether vOLs follow a specific pattern of distribution across all layers of the neocortex. To map the vOL population, we labeled the vasculature with CD31 or collagen-IV and oligodendrocytes and myelin with CNPase (Fig. 3A). Vascular density appeared uniformly distributed (Fig. 3B), whereas oligodendrocytes and myelin density increased with neocortical depth, in line with previous work [27]. The increase in myelin density was positively correlated with an increase in oligodendrocyte cell body density (Fig. 3C), thus oligodendrocyte cell body location and density can be used to infer myelination density.

Next, we determined vOL locations in all neocortical layers (Fig. 3D). Similarly to the overall increase in oligodendrocyte density in deeper layers (Supplementary Fig. 4B and 4D), vOLs increased in occurrence with cortical depth (Fig. 3E and Supplementary Fig. 4C and 4E). As a result, the proportion of vOLs remained stable across all neocortical layers. In the motor cortex, vOLs represented 17.7 ± 1.1% of oligodendrocytes (n = 6 mice, Kruskal-Wallis test p = 0.31, Fig. 3F) and 16.6 ± 1.2% in the somatosensory cortex (n = 6 mice, Kruskal-Wallis test p = 0.36, Fig. 3G) across all neocortical layers. The total percentage of vOLs across the whole cortex was similar in primary motor and somatosensory areas (Mann-Whitney test, p = 0.5, n = 6 mice). The increase of vOL occurrence with distance from the pia was not correlated with the vascular density (Pearson correlation, R^2^ = 0.21, p = 0.1, n = 3 mice). We ruled out that the association of oligodendrocytes with the vasculature was a by chance occurrence as analyzing images, in which the vasculature was horizontally flipped 180º, resulted in a reduced percentage of vOLs (p = 0.033, paired t-test, n = 5 mice, Fig. 3H).

**Fig. 4:**
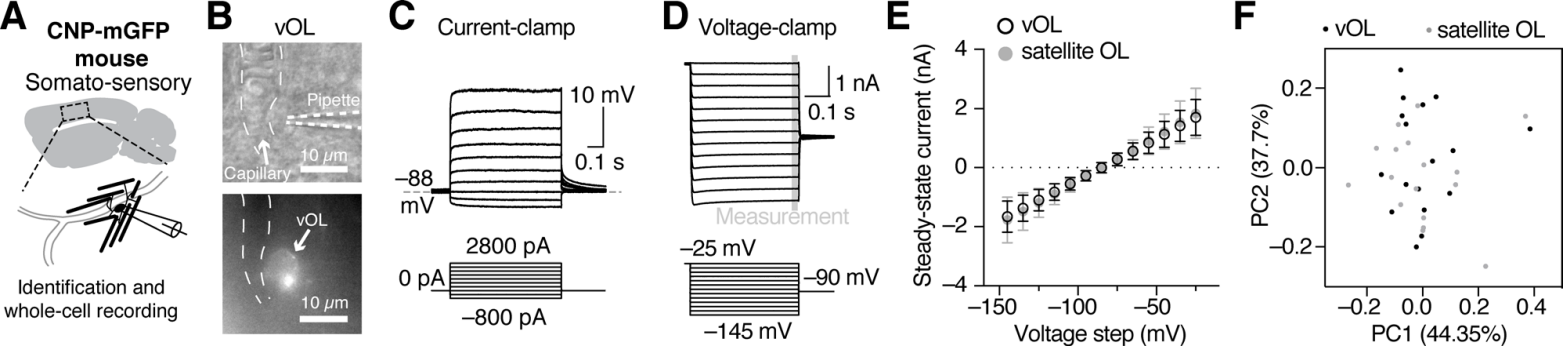
**Physiological analysis of vOLs reveals similar physiological properties as satellite oligodendrocytes.** (A) Schematic of a parasagittal acute slice and the recording location in the somatosensory cortex with a schematic of a vOL. (B) Example image of a vOL with patch-clamp pipette in oblique contrast and the corresponding GFP fluorescence image for identification. (C) Representative example traces from current-clamp whole-cell recordings of a vOL. (D) Example whole-cell voltage clamp recording of a vOL with corresponding command voltages. The steady-state measurement is indicated and displayed in E. (E) Current-voltage (I-V) relationship of vOLs compared to satellite OLs shows a linear I-V relationship for oligodendrocytes from both anatomical locations in the steady-state. (F) Principal component analysis based on the electrophysiological parameters shows no separation of the two groups of oligodendrocytes.

As brain regions differ in their myelination patterns, we examined the presence of vOLs in other brain areas. Analysis of the hippocampal CA1 region including stratum pyramidale, stratum oriens and stratum radiatum and the cell layers of the cerebellar anterior lobe IV/V confirmed the presence of vOLs in both regions (Supplementary Fig. 4F, G). Quantifications showed that vOLs represent 16.6 ± 2.3% of the oligodendrocyte population in the hippocampus (118 out of 678 cells, n = 4 mice) and 20.8 ± 2.8% in the cerebellum (129 out of 611 cells, n = 3 mice, Supplementary Fig. 4H). We conclude from these experiments that vOLs are frequent, and a common component of the vessel micro-environment in the adult brain.

Given the role of oligodendrocytes in potassium buffering [22, 28] and the effect of extracellular potassium in vasodilation [29, 30] we explored intrinsic membrane properties of neocortical vOLs and compared them to satellite oligodendrocytes that are attached to neuronal somas [22]. We performed whole-cell recordings in current and voltage-clamp from identified vOLs and satellite oligodendrocytes in the somato-sensory neocortex (Fig. 4A-D). Resting membrane potential, input resistance, resting conductance and capacity of vOLs and satellite oligodendrocytes were not different between these two subtypes (Table 2). Analysis of the current-voltage relationship showed that both oligodendrocyte subtypes had a similar linear response to command voltages (Kolmogorov-Smirnov test, p = 0.99, Fig. 4D and E). In line with these data, principal component analysis of the electrophysiological parameters showed little separation of the two anatomically distinct oligodendrocyte populations (Fig. 4F). We assessed gap-junction coupling by biocytin diffusion from the recorded vOL to neighboring cells. Both astrocytes (6 ± 2) and oligodendrocytes (7 ± 3) from 3 recorded vOLs were labeled, indicating functional gap junctional coupling between these glial cell types.

**Table 2:** Electrophysiological membrane properties of oligodendrocytes (Mean ± SEM)

Membrane property	Vascular-associated OLs	Satellite OLs	Statistical test and p-value
Resting membrane potential – V_m_ (mV)	–86.47 ± 0.80 (n = 22, n = 14 mice)	–84.41 ± 0.78 (n = 17, n = 16 mice)	unpaired t-test p = 0.08
Input resistance - R_in_ (MΩ)	11.98 ± 2.0 (n = 22, n = 14 mice)	14.74 ± 3.2 (n = 17, n = 16 mice)	Mann-Whitney test, p = 0.66
Resting conductance – G_m_ – (nS)	27.0 ± 1.9 (n = 24, n = 14 mice)	30.94 ± 3.1 (n = 17, n = 16 mice)	unpaired t-test p = 0.25
Capacitance (pF)	62.68 ± 5.3 (n = 19, n = 14 mice)	64.72 ± 5.2 (n = 17, n = 16 mice)	unpaired t-test p = 0.78

In summary, one out of six oligodendrocytes associates with the vasculature across different brain regions. vOLs have a similar electrophysiological profile compared to satellite oligodendrocytes in the gray matter, suggesting that although located at anatomically distinct functional structures these subpopulations are not clearly identifiable by their electrophysiological properties.

### Vasculature-associated oligodendrocytes are regenerated during remyelination

**Fig. 5:**
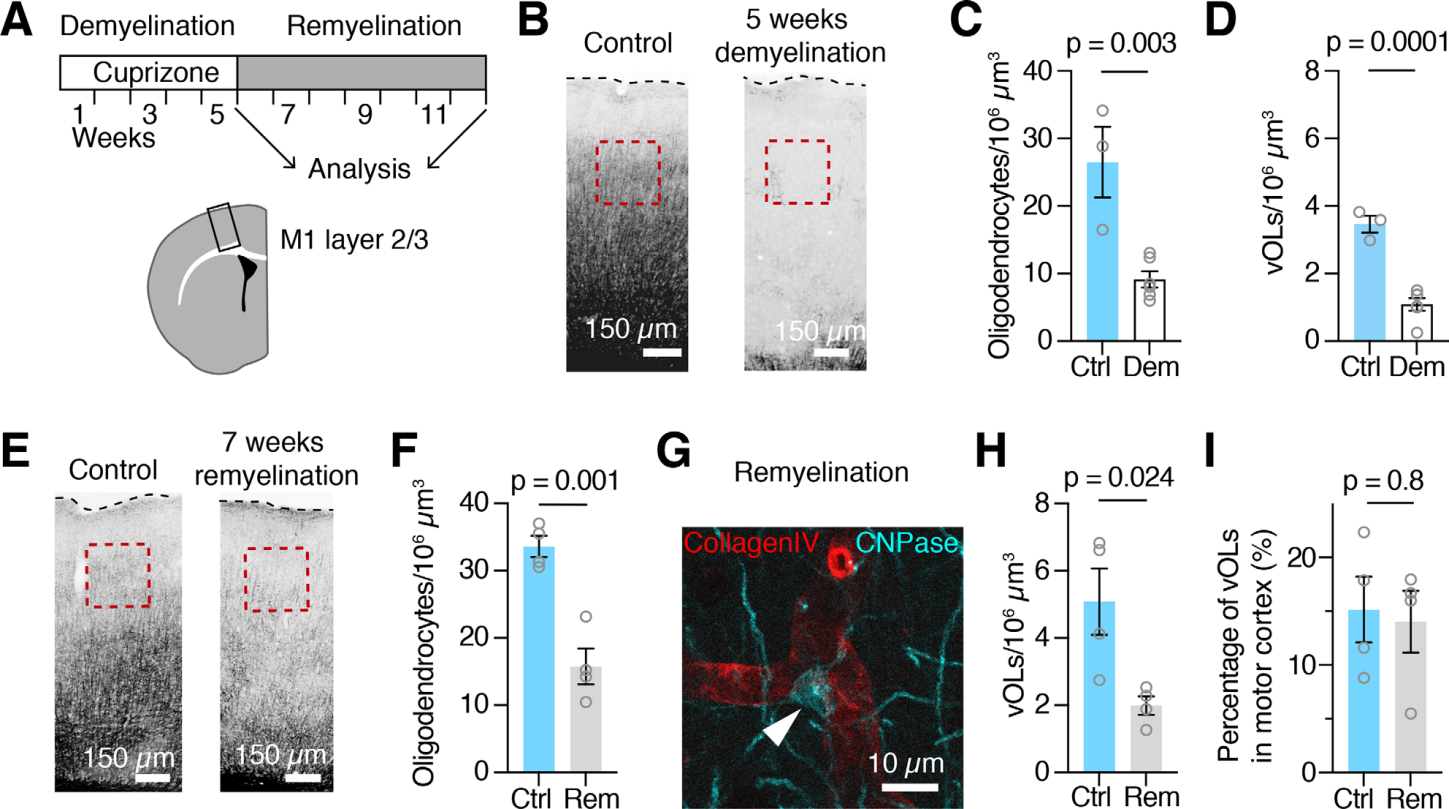
**Remyelination reestablishes vascular association of oligodendrocytes** (A) Schematic of the experimental timeline and schematic overview and locations of the microscopy images shown in B and D (black box). (B) CNPase labeling of the motor cortex reveals that 5 weeks of cuprizone treatment leads to near complete demyelination compared to control. Red boxes indicate the zone of quantification in layer 2/3, a dotted line denotes the cortex surface. (C) Quantifications of the oligodendrocyte density after 5 weeks of demyelination in layer 2/3, compared to age matched controls (12 week old mice, unpaired t-test). (D) Quantifications in layer 2/3 show that vOLs are strongly reduced (unpaired t-test). (E) Example overview images of the cortex from age matched control and remyelinated mice that were labeled with a CNPase antibody. In the remyelinated cortex, myelin still appears less homogeneous compared to control. Red boxes indicate the area of quantification in layer 2/3. (F) Quantifications in layer 2/3 show that after 7 weeks of remyelination, total oligodendrocyte density is still reduced in motor cortex compared to age matched control mice. Age of mice at time of analysis is 22 weeks. Unpaired t-test, p = 0.001. (G) Example of a vOL in motor cortex (arrow head) after 7 weeks of remyelination. (H) Quantifications of vOL density during remyelination show a reduction compared to control. Single data points represent data from one animal. Unpaired t-test, p = 0.024, n = 4 animals for control and for remyelination. (I) Quantifications reveal that the percentage of vOLs during remyelination is comparable to the control motor cortex. Unpaired t-test, p = 0.8, n = 4 animals for control and for remyelination. Single data points in C, D, F, H and I represent averaged data from one animal.

It was previously reported that OPCs associate with the vasculature during remyelination [2] and differentiate to mature oligodendrocytes to compensate for oligodendrocyte loss in the lesion. However, during remyelination the cell bodies of newly formed oligodendrocytes appear in different locations and display a different myelination pattern [31–33]. To assess potential changes of the vOL population during remyelination, mice were fed with cuprizone-supplemented food for 5 weeks to induce demyelination and subsequently returned to normal food for 7 weeks (Fig. 5A). We first analyzed the extent of neocortical demyelination in the motor cortex after 5 weeks of demyelination (Fig. 5B, C). Compared to control mice (n = 3 mice), the cortex of cuprizone-fed mice was almost completely devoid of oligodendrocytes, except for some oligodendrocytes in layer 2/3 and a few oligodendrocytes close to the white matter (n = 6 mice). Quantifying the density of the remaining oligodendrocytes in layer 2/3 showed that after 5 weeks of demyelination, only 9 ± 1 oligodendrocytes per 10^6^ µm^3^ remained compared to 27 ± 5 oligodendrocytes per 10^6^ µm^3^ in control cortex (Fig. 5C, unpaired t-test, p = 0.003). Cuprizone affected vOLs as well, with only 1.1 ± 0.2 vOLs remaining per 10^6^ µm^3^ compared to 3.5 ± 0.3 vOLs per 10^6^ µm^3^ in age matched controls (Fig. 5D). The percentage of vOLs after demyelination, however, remained unchanged (unpaired t-test, p = 0.8). Even though we could not distinguish between new oligodendrocytes formed during demyelination or oligodendrocytes that survived the cuprizone treatment, these results confirmed that cuprizone treatment was highly effective in reducing uniformly all neocortical oligodendrocytes independently of their vascular association.

To investigate whether mature oligodendrocytes associate with the vasculature during remyelination, we analyzed the layer 2/3 of the motor cortex after 7 weeks of remyelination (Fig. 5D). We found that oligodendrocytes in these mice were still reduced by 50 ± 9% (n = 4 mice) compared to age matched control mice (n = 4 mice, Fig. 5E, unpaired t-test, p = 0.001). In the remyelinated cortex vOLs were present (Fig. 5F), but reduced by 63 ± 7% compared to age matched control mice (n = 4 mice in control and in remyelination, Fig. 5G). While the overall number of vOLs was decreased, the proportion of vOLs in the remyelinated motor cortex was comparable to the control cortex (Fig. 5H and I).

Our results show that in the remyelinating motor cortex, vOLs occur in the same proportion as in control mice, suggesting a homeostatic regulation of this oligodendrocyte population.

## Discussion

In this study we show that mature oligodendrocytes frequently and closely associate with the vasculature. As the position of oligodendrocyte cell bodies is very stable during lifetime [70], we suggest that vOLs are a component of the neurovascular unit. Given their intermediate location between blood vessels, axons and other oligodendrocytes, vOLs might play essential roles in central nervous system function.

### Mature oligodendrocyte association with the vasculature

Although the association of oligodendrocytes with blood vessels has been described almost a century ago [10], this observation was only explored or noted in few further studies [11–13]. Here, we present evidence based on (i) immunohistochemistry, and (ii) correlative light and volume EM, that vOLs are common and establish a direct contact with the basement membrane of the vasculature (Figs. 1 and 2).

Chemical fixation and dehydration result in tissue shrinkage and a reduction of the extracellular space around the vasculature [34], suggesting that the cellular arrangement could be more loose (Figs. 1, 2, 3 and 5). However, we consistently found vOLs in living tissue in electrophysiology experiments (Fig. 4). Our data further support the recent proposition that some oligodendrocytes are physically attached to the vasculature, concluded from single-cell/nucleus sequencing data sets obtained from purified vessel-associated cells of mice [13] and human [35]. Moreover, we show that vOLs represent a significant and constant proportion of oligodendrocytes in different brain regions, expanding recent findings of OPC interaction with the vasculature [1, 2, 5, 36] to mature oligodendrocytes.

Oligodendrocytes interact with most cells that have been defined as constituting the neuro-vascular unit: neurons, astrocytes, microglia and OPCs. Our finding that the majority of vOLs associates with capillaries suggests that the cellular arrangement, the molecular composition or diffusible factors like vascular endothelial growth factor A, transforming growth factor-β, or wnt, could play a role in vOL formation and maintenance as it has been shown for OPCs [1, 37].

### Wnt-dependent association of oligodendrocytes with the vasculature

To date, the Wnt signaling pathway is one of the best identified signaling mechanisms that is involved in vasculature-OPC interaction. It has been demonstrated that physical OPC-vasculature interaction relies on Wnt-dependent signaling via the activation of Cxcr4 receptor in OPCs. Inactivation of this pathway, leads to detachment of OPCs from blood vessels and subsequent OPC differentiation [1]. This model is further supported by the recent discovery of OPC perivascular clusters in multiple sclerosis tissue, which has been linked to Wnt pathway overactivation in OPCs [2].

The existence of vOLs is in contrast with these earlier works, as vOLs might be generated from non-detached OPCs. Notably, vOLs were regenerated during remyelination (Fig. 5), further supporting the possibility that vOLs could originate from OPCs that remain on blood vessels. This reasoning could indicate functional heterogeneity in response to Wnt signaling and its various downstream signaling pathways within the oligodendrocyte lineage [38]. Local increases in Wnt may alternatively originate from other sources as astrocytes that release Wnt to maintain endfeet integrity [39]. As a consequence, a sustained Wnt tone from astrocytes might inhibit the detachment of some OPCs. However, as we observe one out of six oligodendrocytes at the vasculature, the proposed model of OPC detachment [1] remains plausible for the majority of OPCs.

Instead, association of oligodendrocytes with the vasculature could also be Wnt-independent, secondary to detachment, or regulated through other factors. Oligodendrocytes could be intrinsically primed to attach to the basement membrane via laminin-α2 (*Lama2*), which is expressed in oligodendrocytes and plays a role in myelination and OPC maturation [13, 40, 41]. Moreover, laminin-α2 has been shown to be required for astrocytic endfeet maintenance and attachment to the basement membrane [42] and might therefore play a similar role in vOLs, but this remains to be further investigated.

### Potential functions of vasculature-associated oligodendrocytes

Oligodendrocytes are heterogeneous (reviewed in [43]) and can be classified based on their developmental origin [44], transcriptomic status [40, 45], anatomical organization [10, 25, 45, 46] or functional impact [22]. Whether the population of vOLs has distinct functional properties remains to be addressed in future studies and we next consider how the direct association of vOLs with the vasculature could influence oligodendrocyte, blood vessel and ultimately neuronal functions.

First, oligodendrocytes provide metabolites, such as lactate and glucose, to neurons to maintain ion gradients during axonal action potential firing [47–50]. In this respect, vOLs are well positioned to directly import glucose from endothelial cells and respond rapidly to increased metabolite demand without an intermediate transfer from astrocytes. Such a metabolic coupling could be particularly relevant in learning conditions in which increased energy demand is revealed by functional hypoxia [51].

Second, oligodendrocytes participate in potassium buffering [22, 28] and given the role of extracellular potassium in pericyte relaxation and subsequent dilation of capillaries [29, 52], vOLs could modulate vascular tone by locally regulating extracellular potassium. Vasculature-associated oligodendrocytes are mainly found on capillaries (Fig. 1) and potassium increase around capillaries has been shown to induce upstream dilation of arterioles in vivo [30]. Notably, we observed that 22% of vOLs are located at vasculature bifurcations (Fig. 1), which could reflect a role in potassium-dependent vasodilation of higher order blood vessels. Moreover, capillary dilation has also been observed in response to a physiological stimulus or glutamate application [53, 54]. Whether vOLs interfere with capillary dilation by modulating extracellular glutamate via glutamate transporter activity [55, 56] remains to be explored.

Third, the existence of a direct contact between vOLs and blood vessels allows a quick response to vasculature derived signals, e.g. the release of endothelin by endothelial cells, which regulates myelin sheath numbers in response to neuronal activity [8]. However, oligodendrocytes might respond to endothelin only during short periods of maturation and myelin formation [8]. Another possibility are pericyte derived signals, as pericyte conditioned media has been shown to increase proliferation of cultured OPCs [57].

Fourth, oligodendrocytes are the brain cells with the highest amount of iron, which is necessary for myelination [58]. The majority of vOLs were located on transferrin receptor positive blood vessels (Fig. 1G,H) and all oligodendrocytes [40, 59] including vessel-associated oligodendrocytes [13] have been found to highly express transferrin, which is required for cellular iron import in the brain. As a result, vOLs may act as an entry point for iron into the gap-junction-coupled oligodendrocyte network.

### Implications in pathologies

Vasculature changes, including alterations of the blood-brain barrier, have been implicated in several neurodegenerative diseases (reviewed [60]). Although vascular dysfunctions have been noted in multiple sclerosis, their relation and impact on disease progression are unclear [61]. Notably, type 2 gray matter lesions often occur around the vasculature [62], where early signs of blood-brain barrier disruption are also found [63]. In the experimental autoimmune encephalomyelitis (EAE) model, oligodendrocytes have been identified to present antigens [64] and altered oligodendrocyte heterogeneity has been found in multiple sclerosis [64, 65]. Another study revealed an increase of OPCs on micro-vessels in the EAE model [37], similar to multiple sclerosis tissue [2], suggesting that proliferation and differentiation around the vasculature could impact the formation of new oligodendrocytes in the vascular niche. We did not observe an increase of vOLs during remyelination, however, analysis of earlier time points in the remyelination process might provide a better insight in the dynamics of vOL regeneration. It remains to be investigated whether vOLs could be more vulnerable than other oligodendrocytes, as vOLs could either be an early target during immune cell infiltration into the brain parenchyma [66] or play a role in initiating immune cell contact as antigen-presenting cells [64]. It is noteworthy that cuprizone induced demyelination leads to a blood-brain barrier permeability increase after a few days of cuprizone treatment [67], thus whether vOLs are more vulnerable could be addressed in longitudinal in vivo studies during disease progression or demyelination in future experiments.

Oligodendrocyte-vasculature interaction has also been shown in white matter injury, where secretion of metalloproteinase-9 by oligodendrocytes promotes angiogenesis [9], a mechanism that would be especially relevant for vOLs. In conclusion, vOLs might contribute to oligodendrocyte vulnerability and vasculature changes in pathological conditions.

## Conclusion

We focused on identifying the fundamental characteristics of vasculature interaction with mature oligodendrocytes. Astrocyte interaction with the vasculature is well established, and recent studies show that microglia and OPCs also interact with blood vessels [1, 5, 68, 69]. Here, we provide evidence that mature oligodendrocytes interact with blood vessels as well, suggesting a general crosstalk of all glial cells with the vasculature.

Our study presents the basis for future studies of vasculature-oligodendrocyte interaction in both physiology and pathology. Vasculature alterations and the susceptibility of oligodendrocytes in several neurodegenerative diseases show the importance of further addressing this intricate cellular interaction.

## Electronic supplementary material

Below is the link to the electronic supplementary material.

Supplementary Material 1

## Data Availability

Further information and reasonable requests for resources and reagents should be directed to the corresponding author (arne.battefeld@u-bordeaux.fr).
