# Supplementary material for: Direct association with the vascular basement membrane is a frequent feature of myelinating oligodendrocytes in the neocortex
Source: Fluids Barriers CNS. 2023 Apr 3;20:24. doi: 10.1186/s12987-023-00425-4 (PMC10069068; doi:10.1186/s12987-023-00425-4)
Supplement: Supplementary file 1 — Supplementary Material 1 [file 12987_2023_425_MOESM1_ESM.pdf]

Supplementary Material for

Direct association with the vascular basement membrane is a frequent feature of myelinating oligodendrocytes in the neocortex

Justine S. C. Palhol, Maddalena Balia, Fernando Sánchez-Román Terán, Mélody Labarchède, Etienne Gontier, Arne Battefeld*

* Corresponding author. Email: arne.battefeld@u-bordeaux.fr

This file includes:

Figures S1-S4

Supplementary Table 1

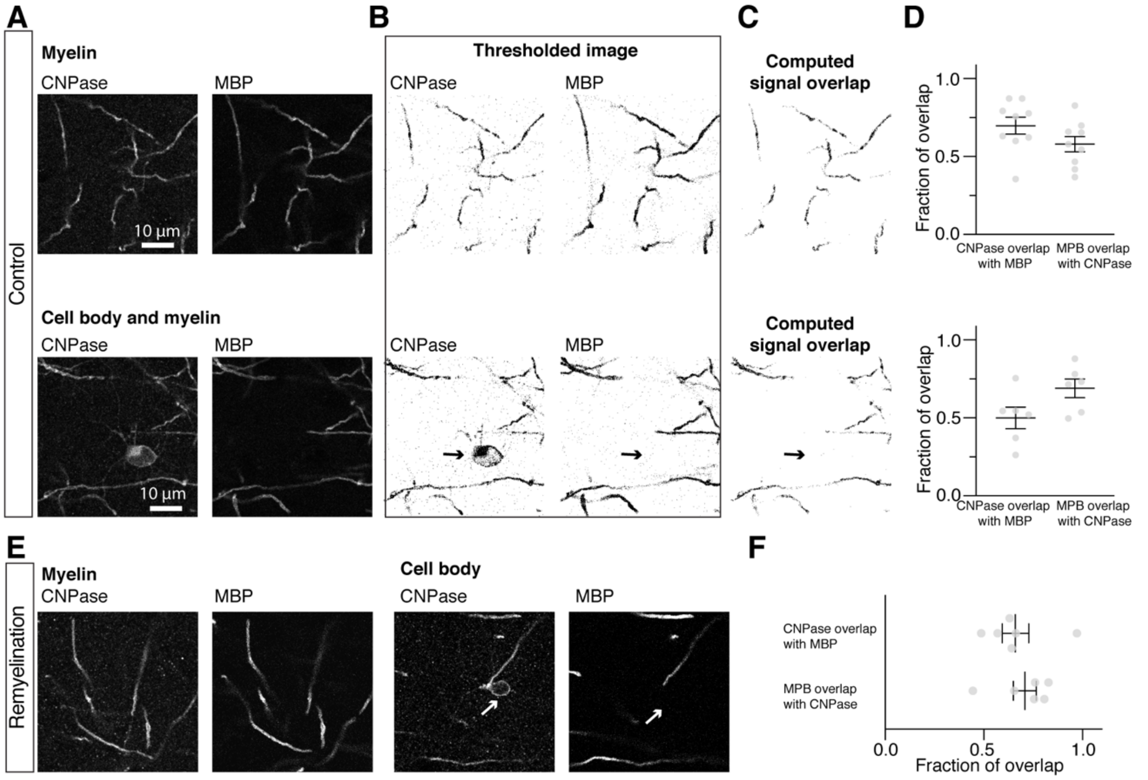

Supplementary Figure 1: Co-labeling to determine antibody specificity for oligodendrocyte structures.

(A) Confocal images of control mouse somatosensory cortex tissue labeled with antibodies against CNPase and MBP. MBP labeling with the used antibody is absent from the oligodendrocyte cell-body. (B) Images were thresholded (mean + 4 x standard deviation) and the overlap of each signal was determined with JACoP (1). (C) We subsequently used FIJI for displaying the calculated pixel overlap of the example images. (D) Quantifications of the overlap fractions for each antibody. For the cell body, the fraction of CNPase overlapping with MBP appears lower. (E) High-magnification confocal images of mouse somatosensory cortex after 5 weeks of demyelination labeled with antibodies against CNPase and MBP. Few myelin sheaths and cell bodies were left, however as in controls cell bodies are only labeled with CNPase. (F) Quantification of the overlap of the two immunosignals after 5 weeks demyelination.

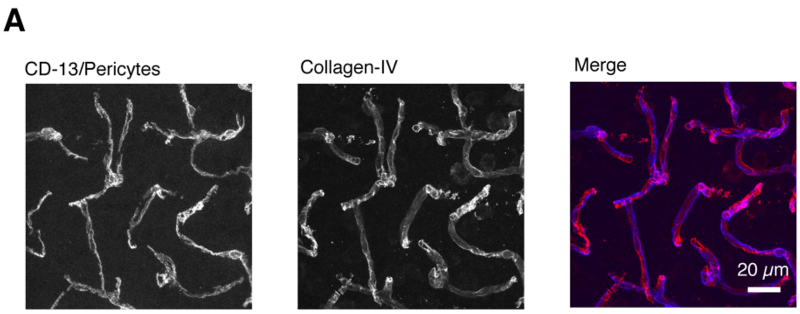

Supplementary Figure 2: Pericytes cover the analyzed cortical vasculature.
(A) Confocal z-projected overview images of CD-13 (pericytes, blue) and collagen-IV (red) demonstrating that the somatosensory cortex vasculature is highly covered by pericytes.

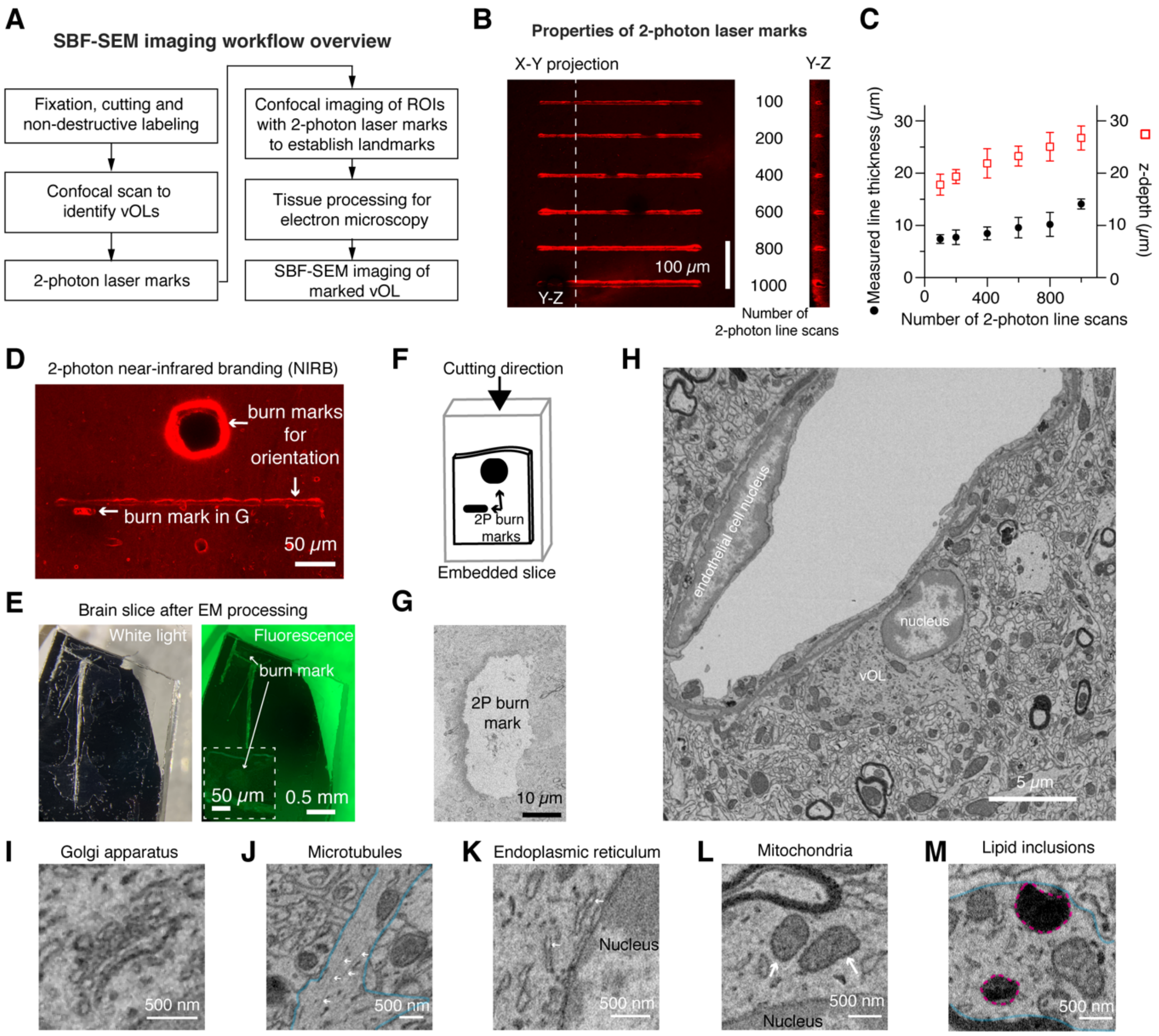

Supplementary Figure 3: SBF-SEM imaging workflow ultrastructure examples (A) Workflow of near-infrared branding guided serial block-face imaging. (B) 2-photon tissue burn lines for estimating the laser power needed to achieve good tissue marking. (C) Quantification of the laser marks thickness and depth, used as reference for subsequent 2-photon laser burns. (D) Example image of laser mark pattern used for final marking. A large squared burn mark was made to identify the ROI location under low magnification. (E) Tissue block after treatment for EM. In ambient light the tissue block is black, prohibiting identification of burn marks. Fluorescent illumination (right) allows identification of the burn mark under binocular observation. The inset shows the burn mark at higher magnification. (F) Schematic of the mounted tissue on the imaging pin. The cutting direction for serial sectioning is indicated. (G) Example of a low resolution backscattered EM image of a small 2-photon branding mark. Small branding marks were used to identify the location of the ROI in EM images. (H) Full field of view of the image shown in Figure 2B without pseudo-coloring. The darker cytoplasm of the

oligodendrocyte is visible in this representation. (I to M) High magnification cut-outs showing typical cell features of oligodendrocytes as indicated in the labels.

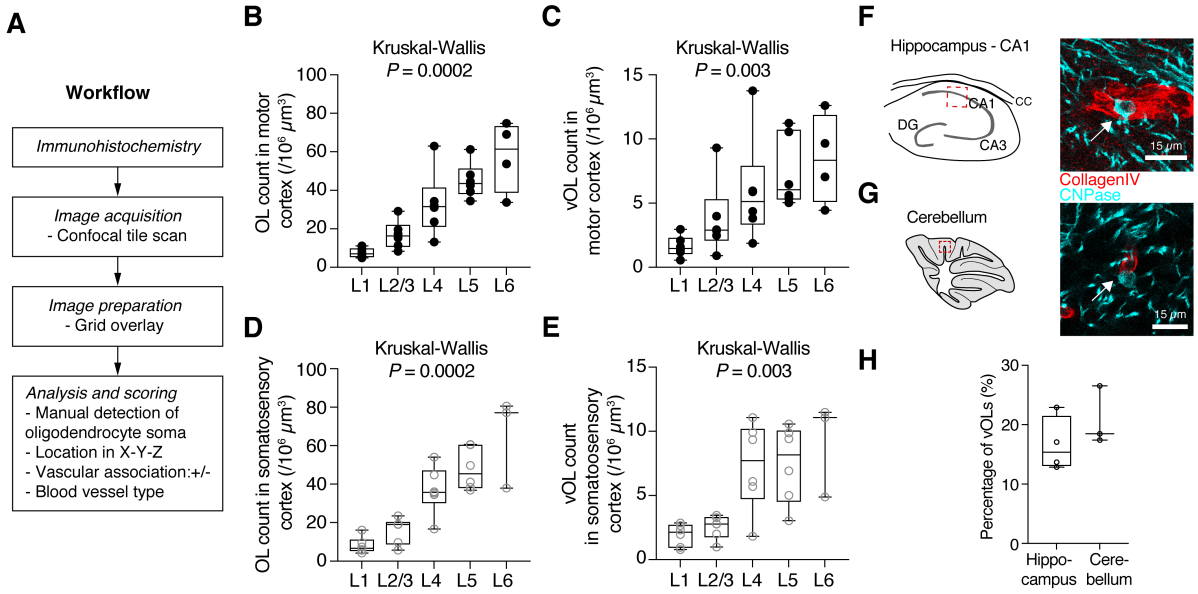

Supplementary Figure 4: Analysis workflow and additional analysis of vOLs

(A) Workflow of immunohistochemistry experiments and subsequent analysis steps to determine cortical oligodendrocyte distribution. (B) Total oligodendrocyte density in each layer of the motor cortex (MC) of 9-week-old mice. Each datapoint represents averaged data from one mouse. Data for CD31 and collagen-IV labeling experiments were summarized. (C) Box plot showing density of vOLs in all cortical layers of the motor cortex. (D) Oligodendrocyte density in the somatosensory cortex of 9-week-old mice. Data points contain vasculature labeling with CD31 and collagen-IV. (E) Quantifications of vOL density in the somatosensory cortex for the different layers. (F) *Left:* Schematic of analysis location in the hippocampal CA1 area. Most oligodendrocytes were located at the axonal projections of CA1 neurons located in the stratum oriens. *Right:* Example of a vOL in the hippocampus (cyan, arrow). (G) *Left*: Schematic of analysis location in the cerebellar Purkinje cell and granule cell layers. *Right:* Example of a vOL in the cerebellum (cyan, arrow). (H) Quantifications of vOLs in the analyzed areas of the hippocampus and cerebellum (hippocampus: n = 4 mice; cerebellum: n = 3 mice).

Supplementary Table 1: Microns dataset cell IDs and information

Name	Cell type	Neuroglancer ID	Bloodvessel ID	Vessel number	Location	# Contact Sites	Vessel size (µm)	Contact type
Pair 1	vOL	864691136296661019	864691134917463050	2	bifurcation	2	7.4	Short stubby contacts
Pair2	vOL	864691135561260996	864691134765900843<br>864691136438598279	2	bifurcation	2	4.2	Short contact
Pair7	vOL	864691136175000838	864691136175000838	1	bifurcation	4	7.7	Short stubby contacts
Pair8	vOL	864691136038074406	864691136534887842	2	bifurcation	2	13.9	Short stubby contacts
Pair9	vOL	864691135993012938	864691136043385126	2	bifurcation	5	6.6	Short stubby contacts

Supplementary References

1. Bolte S, Cordelières FP. A guided tour into subcellular colocalization analysis in light microscopy. J Microsc. 2006;224(3):213–32. Available from: https://doi.org/10.1111/j.1365-2818.2006.01706.x
